# Prevalence of arboviruses in sickle cell disease patients from two major cities in northeast region of Brazil

**DOI:** 10.1016/j.bjid.2024.103741

**Published:** 2024-04-23

**Authors:** José Pereira Moura Neto, Cinthia Cristina Matheus Xerez Albuquerque, Setondji Cocou Modeste Alexandre Yahouedehou, Marcos Vinicius Lima Francisco, Nelson Abrahim Fraiji, Isadora Cristina de Siqueira, Marilda Souza Gonçalves

**Affiliations:** a Fundação Oswaldo Cruz (FIOCRUZ), Instituto Gonçalo Moniz, Salvador, BA, Brazil; b Faculdade de Ciências Farmacêuticas, Laboratório de Análise Especializada em Biologia Molecular (LAEBM), Universidade Federal do Amazonas, Manaus, AM, Brazil; c Fundação Hospitalar de Hematologia e Hemoterapia do Amazonas (HEMOAM), Universidade do Estado do Amazonas (UEA), Manaus, AM, Brazil

**Keywords:** Arbovirus, ZIKV, DENV, CHIKV, Sickle cell disease, Salvador, Manaus

## Abstract

Sickle Cell Disease (SCD) is a hereditary disease characterized by extravascular and intravascular hemolysis and clinical variability, from mild pain to potentially life-threatening. Arboviruses include mainly Zika (ZIKV), Chikungunya (CHKV), and Dengue (DENV) virus, and are considered a public and social health problem. The present cross-sectional observational study aimed to investigate the prevalence of arbovirus infection in SCD patients from two Brazilian cities, Salvador and Manaus located in Bahia and Amazonas states respectively. A total of 409 individuals with SCD were included in the study, and 307 (75.06 %) patients tested positive for DENV-IgG, 161 (39.36 %) for ZIKV-IgG, and 60 (14.67 %) for CHIKV-IgG. Only one individual was positive for DENV-NS1 and another for DENV-IgM, both from Salvador. No individuals had positive serology for ZIKV-IgM or CHIKV-IgM. Arbovirus positivity by IgG testing revealed that the SCD group presented high frequencies in both cities. Interestingly, these differences were only statistically significant for ZIKV-IgG (*p* = 0.023) and CHIKV-IgG (*p* = 0.005) among SCD patients from Manaus. The reshaping of arbovirus from its natural habitat by humans due to disorderly urban expansion and the ease of international Mobility has been responsible for facilitating the spread of vector-borne infectious diseases in humans. We found the need for further studies on arboviruses in this population to elucidate the real association and impact, especially in acute infection. We hope that this study will contribute to improvements in the personalized clinical follow-up of SCD patients, identifying the influence of arbovirus infection in severe disease manifestations.

## Introduction

Arboviruses are transmitted to vertebrate hosts by arthropod vectors. Females of the *Aedes* genus are the vectors that disseminate the arboviruses of primary global clinical importance, i.e., dengue, zika, and chikungunya.^1^ In Brazil, the most important arboviruses belong to the Flavivirus (dengue, zika) and Alphavirus (chikungunya) genera.^2^ In Brazil, in 1845, the first major dengue epidemic was reported in the state of Rio de Janeiro, followed by outbreaks in other Brazilian states between 1851 and 1923.^3^ After a critical campaign to eliminate the *Aedes aegypti* vector, occasional epidemics remained in Brazil until 1976.^4^ Currently, four dengue virus serotypes have been described in Brazil (Dengue virus types 1, 2, 3, and 4). Unfortunately, Brazil is currently suffering major dengue epidemics and has faced the emergence of new arboviruses, such as chikungunya and zika.^5^ Chikungunya fever was first detected in Brazil in September 2014, when the first reports of autochthonous transmission appeared in the state of Amapá, followed by the state of Bahia, leading to a significant increase of more than 700 % in 2016.^6^ The first cases of autochthonous zika virus transmission were recorded in May 2015 in the northern capital of Natal (Rio Grande do Norte, Brazil), with the highest number of cases also recorded in 2016.^7^

Sickle Cell Disease (SCD) is a hereditary disease characterized by the presence of the Hemoglobin S variant (HbS) in association with other hemoglobin variants or thalassemia. Sickle Cell Anemia (SCA) is determined by the Homozygous condition (HbSS).^8^ Symptoms begin in childhood and present intermittently, including moderate to severe anemia, tiredness, pallor, and intense pain crises. Some patients experience complications associated with chronic hemolytic anemia, requiring regular blood transfusions.^9^ In addition to the risk of iron overload and alloimmunization, acute thrombotic events often occur, in some cases potentially lethal, such as vaso-occlusive crises and acute chest syndrome.^10^

The literature contains isolated reports on fatal cases of arbovirus infection in patients with SCD. During the 2001‒2002 epidemic in Havana (Cuba), three fatal cases of dengue were reported in adults with Sickle cell disease.^11^ In 2013, a study in Curacao described two fatal cases involving dengue infection and Sickle cell disease.^12^ In 2015, a retrospective study conducted in the Caribbean reported four fatal cases in patients with dengue and FD.^13^ In 2016, Zika infection was identified in a female patient with HbSC who died in Colombia^14^ and, most recently (2021), a pregnant woman with Sickle cell disease died after contracting Chikungunya infection and was reported in Puerto Rico.^15^ However, no studies have attempted to investigate the prevalence of arbovirus infection in SCD.

Infection, whether fungal, bacterial, or viral, in patients with Sickle cell disease can lead to adverse clinical events, such as severe hemolytic anemia splenic sequestration, which have been frequently described in the literature. Since we do not have epidemiological data on arboviruses in the population of patients with sickle cell anemia, both of which are very prevalent in the north and northeast of Brazil, we were interested in determining the prevalence of antibodies against dengue, chikungunya and zika viruses in populations of SCD patients residing in two Brazilian capitals, Salvador (Bahia) and Manaus (Amazonas).

## Methods

The present cross-sectional observational study was conducted in two cities in northern/northeastern Brazil, between May and October 2021.The participants were recruited in reference outpatient clinics, at the Bahia State Hematology and Hemotherapy Foundation (HEMOBA) from the city of Salvador, the capital of the state of Bahia, and the Amazonas State Hematology and Hemotherapy Foundation (HEMOAM), in the capital city of Manaus (Amazonas). Inclusion criteria confirmed by high-performance liquid Chromatography and Real-time PCR (qPCR). Patients were randomly selected for participation, and upon inclusion, patients were interviewed, and clinical history information was obtained from medical records.

Participants were enrolled consecutively during their outpatient care, which was carried out periodically at HEMOBA and HEMOAM, according to the Guidelines of the National Policy for Comprehensive Care for People with Sickle Cell Disease implemented by the Ministry of Health of Brazil.

After the inclusion of the participant, an individual clinical form was generated containing personal information (identification, age, gender, family history) and medical information (hemoglobin genotype, hospitalizations, transfusions, clinical manifestations, surgeries) according to information collected during interviews with patients and through medical records.

Serological diagnosis for arboviral infection was performed by Enzyme-Linked Immunosorbent Assays (ELISA) using EUROIMMUN (Lüberg, Germany) kits with solid-phase antigens coated on 96-well polystyrene plates. Diagnostic assays were performed using the following kits: DENV-NS1: (Dengue Nonstructural one glycoprotein); DENV-IgG: (Dengue specific Immunoglobulin G); DENV-IgM (Dengue specific Immunoglobulin M); ZIKV-IgG (Zika specific Immunoglobulin G);^16^ ZIKV-IgM (Zika specific Immunoglobulin M);^17^ CHIKV-IgG (Chikungunya specific Immunoglobulin G) and CHIKV-IgM (Chikungunya specific Immunoglobulin M).^18^

The Multiskan™ FC microplate photometer from Thermo Scientific™ was used to conduct ELISA plate readings. Absorbance readings at 450 nm with a reference filter of 620 nm were performed. Results were obtained by calculating the ratio between the optical density of each patient sample compared to that of the calibrator supplied with the kit, in which samples with a ratio < 0.8 were considered negative, samples between ≥ 0.8 to < 1.1 were considered borderline, and samples with a ratio ≥1.1 were considered positive. All samples with initial borderline results were retested.

Statistical analysis was performed using IBM SPSS software, v.19.0 (IBM Corp., Armonk, NY, USA). ANOVA parametric testing was used to analyze the distributions of quantitative variables (expressed as means) with normal distribution within categories. Qualitative and categorical variables were compared among three or more groups using the non-parametric Chi-Squared test (χ2) test with Yates' continuity correction.

The present study protocol was approved by the institutional review board of the Gonçalo Moniz Institute (Fiocruz-BA; CAAE no. 40968120.3.0000.0040) and by the board for ethical research of HEMOAM (CAAE no. 87700518.7.0000.0009). All procedures described herein were conducted following the ethical standards of relevant committees on human experimentation (institutional and national) as well as the Helsinki Declaration of 1975, and its subsequent revisions.

## Results

A total of 409 individuals with SCD participated in the study: 223 from Salvador and 186 from Manaus. In general, females predominated, with 78/46.7 % and 91/55.8 % diagnosed with HbSS from Salvador and Manaus, respectively, versus 29/51.8 % and 14/60.9 % with HbSC. Genetic admixture was prevalent, with most patients self-reporting mixed-race (*pardo*) in both cities. The mean age of patients was slightly higher in Manaus; of note, age at the time of diagnosis was almost 12-fold higher in patients from Manaus compared to Salvador. Weight and height were similar among SCD patients from both cities (Table 1).

**Table 1:** Demographic characteristic of Sickle cell disease patients from Salvador-BA and Manaus-AM, Brazil.

City	Salvador (*n* = 223) (%)		Manaus (*n* = 186) (%)		Total (*n* = 409) (%)	
Genotype	SS	SC	SS	SC	SS	SC
Gender						
Male	89 (53.3)	27 (48.2)	72 (44.2)	9 (39.1)	161 (48.8)	36 (45.6)
Female	78 (46.7)	29 (51.8)	91 (55.8)	14 (60.9)	169 (51.2)	43 (54.4)
Skin color						
White	10 (6.0)	0	9 (5.4)	5 (21.7)	19 (5.7)	5 (6.3)
Brown	96 (57.5)	31 (55.4)	126 (77.6)	15 (65.2)	222 (67.3)	46 (58.2)
Black	61 (36.5)	25 (44.6)	28 (17.0)	3 (13,1)	89 (27.0)	28 (35.5)
Age	13.1 ± 4.6	14.2 ± 2.8	17.6 ± 13.18	22.2 ± 12.8	‒	‒
Age on diagnosis	0.58 ± 0.67	0.63 ± 0.12	7.2 ± 10.5	7.5 ± 9.6	‒	‒
Height in cm	146.1 ± 27.2	159.4 ± 11.3	144.5 ± 23.6	147.4 ± 26.1	‒	‒
Weight in Kg	39.47 ± 14.9	49.9 ± 14.5	41.3 ± 16.6	49.1 ± 20.2	‒	‒

N, Patient count; SS, Sickle cell anemia; SC, Sickle Cell disease.

Most patients resided in their respective state capitals: 196 (87.9 %) were from Salvador versus 40 (12.1 %) from the countryside of the state of Bahia, while 175 (94.1 %) were from the capital city of Manaus and 11 (5.9 %) resided outside the metropolitan area of Manaus.

A total of 307 (75.06 %) patients tested positive for DENV-IgG, 161 (39.36 %) for ZIKV-IgM, and 60 (14.67 %) for CHIKV-IgG. Only one individual was positive for DENV-NS1 and another for DENV-IgM, both from Salvador. No individuals had positive serology for ZIKV-IgM or CHIKV-IgM. The frequency of arboviral antibodies was higher in SCD patients from Salvador compared to Manaus, approximately 10 % higher for DENV-IgG (79.52% vs. 69.35 %) and ZIKV-IgG (43.95% vs. 33.87 %), yet only slightly higher for CHIKV-IgG (15.25% vs. 13.98%), respectively (Table 2).

**Table 2:** Results of the arbovirus serological of Sickle cell disease patients from Salvador, BA and Manaus, AM, Brazil.

Arbovirus	Salvador, 223 (%)			Manaus, 186 (%)			Total (Positives), 409 (%)
	Positive	Negative	Borderline	Positive	Negative	Borderline	
	(O.D ratio ≥ 1)	(O.D ratio < 0.8)	(O.D ratio ≥ 0.8 to < 1.1)	(O.D ratio ≥ 1)	(O.D ratio < 0.8)	(O.D ratio ≥ 0.8 to < 1.1)	
DENV-NS1	1 (0.45)	222 (99.55)	0	0	186 (100.0)	0	1 (0.24)
DENV-IgM	1 (0.45)	222 (99.55)	0	0	184 (98.9)	2 (1.1)	1 (0.24)
DENV-IgG	178 (79.82)	40 (17.94)	5 (2.24)	129 (69.35)	49 (26.34)	8 (4.30)	307 (75.06)
ZIKV-IgM	0	223 (100.0)	0	0	186 (100.0)	0	0
ZIKV-IgG	98 (43.95)	117 (52.47)	8 (3.59)	63 (33.87)	118 (63.44)	5 (2.69)	161 (39.36)
CHIKV-IgM	0	223 (100.0)	0	0	186 (100.0)	0	0
CHIKV-IgG	34 (15.25)	183 (82.06)	6 (2.69)	26 (13.98)	158 (84.95)	2 (1.07)	60 (14.67)

DENV-NS1, Dengue Nonstructural 1 glycoprotein; DENV-IgG, Dengue specific Immunoglobulin G; DENV-IgM, Dengue specific Immunoglobulin M; ZIKV-IgG, Zika specific Immunoglobulin G; ZIKV-IgM, Zika specific Immunoglobulin M; CHIKV-IgG, Chikungunya specific Immunoglobulin G; CHIKV-IgM, Chikungunya specific Immunoglobulin M; O.D, Optical Density.

Arbovirus positivity by IgG testing revealed that the SCD group presented higher frequencies in both cities. Interestingly, these differences were only statistically significant for ZIKV-IgG (*p* = 0.023) and CHIKV-IgG (*p* = 0.005) in SCD patients from Manaus (Table 3).

**Table 3:** Correlation between serology for arbovirus and hemoglobin genotype of SCD patients from Salvador-BA and Manaus-AM, Brazil.

Arbovirus	Hemoglobin genotype	Salvador, n (%)		*p*-value	Manaus, n (%)		*p*-value
		Positive (O.D ratio ≥ 1)	Negative (O.D ratio < 0.8)		Positive (O.D ratio ≥ 1)	Negative (O.D ratio < 0.8)	
DENV-IgG	SS	131 (80.9)	31 (19.1)	0.610	112 (71.8)	44 (28.2)	0.799
	SC	47 (83.9)	9 (17.1)		17 (77.3)	5 (26.7)	
ZIKV-IgG	SS	70 (43.8)	90 (56.2)	0.433	51 (31.7)	110 (68.3)	0.023^a^
	SC	28 (50.9)	27 (49.1)		12 (60.0)	8 (40.0)	
CHIKV-IgG	SS	21 (13.0)	141 (87.0)	0.116	18 (11.1)	144 (88.9)	0.005^a^
	SC	13 (23.6)	42 (26.7)		8 (36.4)	14 (63.6)	

DENV-IgG, Dengue specific Immunoglobulin G; ZIKV-IgG, Zika specific Immunoglobulin G; CHIKV-IgG; Chikungunya specific Immunoglobulin G; SS, Sickle cell anemia; SC, Sickle cell disease; O.D, Optical Density.

a Student's *t*-test (Yates correction).

During the research period, a total of six patients died (5 SS and 1 SC), all from Manaus. However, no deaths were associated with arbovirus infection.

## Discussion

Currently, it is estimated that 400,000 children with hemoglobin variants are born worldwide annually. ^19^ The S allele is most frequently reported in Brazil, affecting nearly 45,000 individuals, with a notably higher prevalence in the northeastern region of the country. Around 0.3 % of the entire population is affected, especially those of African ancestry, with studies demonstrating increasing rates among Caucasians.^20^ Studies involving SCD patients in Brazil typically demonstrate similar frequencies between genders, despite slightly higher numbers of females. Accordingly, the frequencies reported in our study (51.2 % in SS and 54.4 % in SC females) corroborate those reported by other Brazilian studies.^21^^,^^22^

In Manaus, the higher average age (> 7-years) at the time of diagnosis may be due to geographic characteristics inherent in the state of Amazonas, which, due to great distances between municipalities, can hinder patient access to health services.^23^ Additionally, the state of Amazonas has, to date, unfortunately not yet implemented neonatal screening for SCD. By contrast, in Salvador, newborn screening is regularly performed, which likely explains the discrepancy in age at the time of diagnosis (< 6 months).^24^

Growth and weight in SCD patients are delayed compared to individuals with normal Hemoglobin (HbAA). The longitudinal assessment of height and weight in affected patients aids in routine monitoring and early detection of frequent clinical abnormalities. The average patient height and weight reported herein corroborate parameters reported by other national and international studies.^25^, ^26^, ^27^

The worldwide dissemination of arboviral diseases is of much concern to public health. The reshaping of natural habitats by humans, disorderly urban expansion, and the ease of international mobility may all have been responsible for facilitating the spread of vector-borne infectious diseases in humans. ^28^^,^^29^ In Brazil, the neglected status of arboviral infection precludes the detection of outbreaks by public authorities. According to the Pan-American Health Organization, Brazil has registered the highest number of cases of dengue in the world, accounting for almost 70 % of all cases on the planet.^30^ The first case of local CHIKV transmission was reported in the Caribbean in 2013. In Brazil, the first case appeared in Oiapoque, Amapá in 2014, and this state is currently responsible for the most cases in the country, while numbers are growing in Pernambuco and Bahia.^31^^,^^32^ Regarding Zika infection, although the first human cases reportedly occurred in the states of Bahia and Rio Grande do Norte, currently autochthonous ZIKV transmission occurs in all Brazilian states.^33^, ^34^, ^35^

The present study sought to investigate the prevalence of the three most frequent and clinically important arboviral infections in Brazilian individuals with SCD. In addition, we attempted to verify differences in prevalence between the northern and northeastern regions of the country by comparing rates between the cities of Manaus (Amazonas) and Salvador (Bahia). Due to the relatively recent occurrence of ZIKV and CHIKV outbreaks in Brazil, most studies have focused on clinical associations between SCD and dengue infection.

We identified two studies reporting fatal outcomes in SCD patients who contracted dengue fever. Limonta et al.^11^ reported two deaths in patients with SCD in Havana-Cuba during the 2011 and 2022 epidemics, while Moesker et al.^12^ reported two SCD patients (one HbSS and one HbSC) in Curacao. Two other studies reported cases of arboviral infection in SCD that did not result in death. Iversen et al.^36^ described a case of triple-disease manifestation in a 34-year-old Tanzanian woman with SCA (HbSS) with concurrent malaria and dengue infection. Oliveira et al. ^37^ first reported an association between Sickle cell intrahepatic cholestasis and dengue fever in two female patients from Minas Gerais (Brazil).

A single fatal case involving Zika Virus was reported by Arzuza-Ortega et al.^14^ in a 15-year-old girl from Malambo-Colombia. She had SCD, no previous history of hospitalization or episodes of vaso-occlusive crises and never had dengue or chikungunya infection. Following the confirmation of the Zika virus by specific real-time RT-PCR and despite intensive treatment, the patient died 37 h later. The autopsy revealed hepatic necrosis and severe functional asplenia, with multiple drepanocytes and splenic sequestration.

Another single fatal case involved CHIKV in a 21-year-old, 38-week pregnant female with SCD. It is worth mentioning, that neither CHIKV RNA nor antigen were detected in fetal serum or tissue. ^15^

Rankine-Mullings et al. (2015) ^13^ demonstrated the occurrence of confirmed deaths associated with dengue fever in the proportion of 4.1 per 1000 in the general population, while 125 per 1000 in SCD patients (SC) (0.41% vs. 12.5 % respectively). In the same study, was reported a higher case fatality rate of 222/1000 in SC vs. 45/1000 in SS patients; however, it was not statistically significant (*p* = 0.09). The present study corroborated with Rankine-Mullins et al., which showed the highest frequencies for positive IgG in the SCD group. Meantime, it was found significant associations between ZIKV-IgG and CHIKV-IgG, even though the six deaths that occurred in our study, were not associated with arbovirus infection.

Deoxygenation and polymerization of HbS result in reduced solubility and membrane flexibility, high levels of oxidative stress markers, cell membrane damage, and erythrocyte dehydration. The dehydration in SCD patients normally is based on *K*+ loss and a greater propensity for dense cell formation when compared to SS patients. This could be the reason why SCD patients are more susceptible to infection once these red blood cells are more prone to dehydration.^38^, ^39^, ^40^

Although currently, ZIKV infections are less frequent, DENV and CHIKV infections are still quite prevalent in Brazil, and we believe that the identification of circulating blood microparticles may be one of the most efficient approaches for monitoring the clinical magnitude of arbovirus infections in SCD patients.

We emphasize the results obtained in the present study, especially in arbovirus infections in localities with a high prevalence of SCD, as we understand that we must value all infections and be aware of the association between infections and clinical complications that can be fatal. Based on these facts, we carried out a study of the prevalence of antibodies to DENV, ZIKV, and CHIKV in SCD patients.

One limitation of this study is that we didn't perform serological neutralization tests to access the possible cross-reactivity between Dengue and Zika antibodies. However, our frequency of anti-Dengue and anti-Zika IgG antibodies are to other local studies that performed ELISA and Plaque Reduction Neutralization antibodies. ^41^ Also, is worth noting that patients with SCD, generally, have a severe clinical course, with acute and chronic events. However, we do not have information regarding acute episodes of arbovirus, since the participant inclusion and sample collection were carried out at the time of the patient's routine consultation, which made accessing information regarding the acute episode difficult, but allowed us to evaluate the occurrence of previous arbovirus infections.

## Conclusion

This work demonstrated that the population with SCD has a high rate of previous exposure to arboviruses, which leads us to suggest the need for prevention measures among this population group, in addition to greater attention into possible new epidemics, since these patients have severe genetic disease, and there are already reports of deaths in this population due to arbovirus infection. Finally, we found the need for further studies on arboviruses in this population to elucidate the real association and impact, especially in acute infection.

We hope that this study will contribute to improvements in personalized clinical follow-up in SCD patients, identifying the influence of arbovirus infection in severe manifestations of this disease.

## Conflicts of interest

The authors declare that the research was conducted in the absence of any commercial or financial relationships that could be construed as a potential conflict of interest.

## Data Availability

All our data are deposited in a public bank, in which my research group owns. Can make it available whenever requested upon application to my E-mail: jpmn@ufam.edu.br.
